# Guizhi Fuling Wan, a Traditional Chinese Herbal Formula, Sensitizes Cisplatin-Resistant Human Ovarian Cancer Cells through Inactivation of the PI3K/AKT/mTOR Pathway

**DOI:** 10.1155/2016/4651949

**Published:** 2016-05-18

**Authors:** Li Han, Xiaojuan Guo, Hua Bian, Lei Yang, Zhong Chen, Wenhua Zang, Jingke Yang

**Affiliations:** ^1^Zhang Zhongjing College of Chinese Medicine, Nanyang Institute of Technology, 80 Changjiang Road, Nanyang 473004, China; ^2^College of Pharmaceutical Science, Soochow University, 199 Ren-ai Road, Suzhou 215123, China; ^3^Affiliated Cancer Hospital, Zhengzhou University, Dongming Road 127, Zhengzhou 450008, China

## Abstract

The aim of the study was to explore the possible mechanisms that Guizhi Fuling Wan (GFW) enhances the sensitivity of the SKOV3/DDP ovarian cancer cells and the resistant xenograft tumours to cisplatin. Rat medicated sera containing GFW were prepared by administering GFW to rats, and the primary bioactive constituents of the sera were gallic acid, paeonol, and paeoniflorin analysed by HPLC/QqQ MS. Cell counting kit-8 analysis was shown that coincubation of the sera with cisplatin/paclitaxel enhanced significantly the cytotoxic effect of cisplatin or paclitaxel in SKOV3/DDP cells. The presence of the rat medicated sera containing GFW resulted in an increase in rhodamine 123 accumulation by flow cytometric assays and a decrease in the protein levels of P-gp, phosphorylation of AKT at Ser473, and mTOR in a dose-dependent manner in SKOV3/DDP cells by western blot analysis, but the sera had no effect on the protein levels of PI3K p110*α* and total AKT. The low dose of GFW enhanced the anticancer efficacy of cisplatin and paclitaxel treatment in resistant SKOV3/DDP xenograft tumours. GFW could sensitize cisplatin-resistant SKOV3/DDP cells by inhibiting the protein level and function of P-gp, which may be medicated through inactivation of the PI3K/AKT/mTOR pathway.

## 1. Introduction

Ovarian cancer (OC) is the most lethal of the gynaecological malignancies, largely due to the concealed pathogenesis of OC and advanced stage (FIGO stages III and IV) at diagnosis in most patients [1]. The standard of care in the initial chemotherapeutic management of advanced OC is the combination of a platinum agent (carboplatin or cisplatin) and a taxane agent (paclitaxel or docetaxel) given intravenously [2]. Most ovarian cancer patients will respond to initial chemotherapy, but the long-term survival remains poor as a result of recurrence and drug resistance, leading to a 5-year survival rate of 46% [3]. Stordal et al. demonstrated that the recurrence/drug resistance in OC patients treated with first-line platinum/taxane chemotherapy was related to the overexpression of P-glycoprotein (P-gp) [4]. Other studies have shown that the main mechanism of drug resistance was also due to P-gp expression in doxorubicin-, vincristine-, and paclitaxel-resistant OC cell lines [5] and in OC patients [6]. Because P-gp overexpression plays an important role in the recurrence or drug resistance in OC chemotherapy, it is a problem that should be solved quickly to identify P-gp inhibitors and then improve the chemotherapy sensitivity for OC patients.

Unfortunately, there are no agents currently available to “block” P-gp-mediated resistance in the clinic, although four generations of P-gp inhibitors have been developed, and most of them have shown considerable* in vitro* success. The failure may be attributed to nonspecific toxicity, adverse drug interaction, and numerous pharmacokinetic issues associated with the use of P-gp inhibitors in the conducted clinical trials [7]. Thus, novel P-gp inhibitors with proven efficacy and minimal toxicity and/or adverse effects are urgently required to overcome P-gp-mediated resistance in cancer chemotherapy.

Guizhi Fuling Wan (GFW; Gyejibokryeong-hwan in Korean and Keishi-bukuryo-gan in Japan) is a well-known traditional Chinese herbal formula, comprising the five herbs* Ramulus Cinnamomi cassiae*,* Scierotium Poriae cocos*,* Radix Albus paeoniae Lactiflorae*,* Cortex Radicis moutan*, and* Semen Pruni persicae*. GFW originally appeared in* Essential Prescriptions from the Golden Cabinet* (Jinkui Yaolue), a classic clinical book of traditional Chinese medicine (TCM), written by Zhang Zhongjing (150–219 AD) at the end of the Eastern Han Dynasty. GFW has been used extensively throughout Asia in the treatment of blood stasis, and it has been proven to be very safe and effective with fewer harmful side effects. Modern medical research has shown that GFW has various therapeutic effects on conditions such as inflammatory skin disorders [8], endometriosis [9], diabetes-mellitus-induced neuropathology [10], and atherosclerosis [11]. GFW capsules are currently being assessed for the efficacy, safety, and dose-response in the treatment of primary dysmenorrhea by the USA Food and Drug Administration (FDA) [12, 13]. In the recent years, it was reported that catechin, one of the chemical constituents of GFW, is a potent P-gp inhibitor [14]. Moreover, it was demonstrated that the main bioactive constituents of GFW in rat plasma include gallic acid, amygdalin, albiflorin, paeoniflorin, paeonol, cinnamic acid, dehydrotumulosic acid, tumulosic acid, and polyporic acid C [15, 16], while gallic acid, paeonol, and dehydrotumulosic acid were found to have a potent P-gp inhibition effect on multidrug resistance (MDR) cell lines [17–19], and paeoniflorin was found to modulate MDR of the human gastric cancer cell line SGC7901/vincristine (VCR) effectively at nontoxic concentrations via inhibiting the activation of NF-kappaB and subsequently downregulating its target genes* MDR1*, which encodes P-gp [20]. Therefore, accumulating studies have shown that GFW may be a formula demonstrating a P-gp inhibition effect, a finding that has not yet been reported elsewhere.

Recently, more and more researchers have come to realize that drug resistance is a complex phenomenon involving multiple mechanisms, including the activation of multiple signalling pathways. Growing evidence has suggested that the phosphatidylinositol 3-kinase (PI3K)/AKT/mammalian target of rapamycin (mTOR) pathway, wherein* MDR1* and NF-kappaB are downstream factors of this pathway, is closely related to the occurrence of drug resistance in cancer [21–23]. It was also found that cisplatin resistance to OC was related to the PI3K/AKT/mTOR pathway [22]. However, the effect of GFW on the pathway has never been explored.

The aims of the present experiments were to investigate whether GFW has inhibitory effects on P-gp and to identify the underlying molecular mechanisms involving the PI3K/AKT/mTOR pathway in human MDR OC cell lines, SKOV3/DDP, and mouse xenograft model generated from SKOV3/DDP cells.

## 2. Materials and Methods

### 2.1. Plant Materials and Preparation of the GFW Extract

The five herbals forming GFW—that is,* Ramulus Cinnamomi cassiae* (Batch number 201409),* Scierotium Poriae cocos* (Batch number 201410),* Radix Albus paeoniae Lactiflorae* (Batch number 201410),* Cortex Radicis moutan* (Batch number 201409), and* Semen Pruni persicae* (Batch number 201408)—were purchased from the Zhang Zhongjing Pharmacy Chain-Like Limited Company (Nanyang, China). The origin of these herbals was taxonomically double confirmed by Professor Xianzhang Huang, a plant taxonomist from the Zhang Zhongjing College of Chinese Medicine, Nanyang Institute of Technology, where the voucher specimens were deposited.

GFW was prepared in accordance with the process as stated in the Chinese Pharmacopeia (2010 Edition) [24]. Briefly,* Cortex Radicis moutan* was submerged in water and submitted to water distillation (WD) for 1 h with a standard apparatus for extracting volatile oil. The dregs of* Cortex Radicis moutan* were put together with* Ramulus Cinnamomi cassiae, Scierotium Poriae cocos, Radix Albus paeoniae Lactiflorae,* and* Semen Pruni persicae* and then were extracted twice (60 min per time) by refluxing with 90% ethanol (EtOH). The EtOH extracts were combined, filtered, and evaporated under reduced pressure to the appropriate volume. The dregs of the five herbs were extracted subsequently twice (60 min per time) with water. The water extracts were combined, filtered, and evaporated under reduced pressure to the appropriate volume. The concentrated EtOH extract was next combined with the water extract and volatile oil uniformly, and the concentration was adjusted to 1.5 g/mL corresponding to the crude herbs. The mixed solution was used for animal studies and preparing the rat medicated sera.

### 2.2. Cell Culture

The human OC SKOV3 cell line and its cisplatin-resistant SKOV3/DDP cell line were obtained from the Chinese Academy of Medical Sciences and Peking Union Medical College. The cells were cultured in RPMI 1640 (Gibco BRL, Grand Island, NY, USA) supplemented with 10% (v/v) foetal bovine sera (FBS; Hyclone, Logan, UT, USA) at 37°C in a humidified atmosphere containing 5% CO_2_. To maintain resistance, SKOV3/DDP cells were cultured in medium containing 1 *μ*g/mL cisplatin (Qilu Pharmaceutical Co., Ltd., Shandong, China).

### 2.3. Preparation of Rat Medicated Sera

The rat medicated sera were prepared according to the published protocols [25]. Briefly, 60 Wistar female rats, aged between 6 and 8 weeks old and weighing 220~250 g, were randomly divided into four groups (15 rats per group): the control group was given normal saline by gavage, and low-dose (LD) GFW group, middle-dose (MD) GFW group, and high-dose (HD) GFW group were given the mixed GFW solution by gavage at dosages of 4 g·kg^−1^·d^−1^, 8 g·kg^−1^·d^−1^, and 16 g·kg^−1^·d^−1^, respectively. The animals were supplied by the Henan Experimental Animal Center (Zhengzhou, China) and maintained in an air-conditioned room with a controlled temperature of 22 ± 2°C, a humidity level of 45% to 65%, and a 12-/12-hour light/dark cycle. After 5 days of administration, the rats were anaesthetized, and the blood was collected from the abdominal aorta and centrifuged. The collected sera were aliquoted into 1 mL Eppendorf tubes and preserved at −80°C for future use. This study was carried out in strict accordance with the recommendations in the Guide for the Care and Use of Laboratory Animals of the National Institutes of Health. The protocol was approved by the Committee on the Ethics of Animal Experiments of the Nanyang Institute of Technology (Permit number: 2014-L38). The whole surgery was performed under sodium pentobarbital anaesthesia, and all efforts were made to minimize suffering.

### 2.4. Bioactive Constituents of GFW Analysis by HPLC/QqQ MS

An aliquot of 100 *μ*L of LD sera was pipetted into a 1.5 mL capped polypropylene tube, 400 *μ*L of methanol was added, and the contents were mixed by vortexing for 30 s. After centrifuging at 12,000 ×g for 5 min, 400 *μ*L of the supernatant was transferred to a 10 mL glass tube and evaporated to dryness under a steam of nitrogen at +50°C. The residue was redissolved with 100 *μ*L of the mobile phase and briefly vortexed. After centrifuging at 13,000 ×g for 5 min at room temperature again, the supernatant was transferred into a glass autosampler vial and 20 *μ*L aliquot of supernatant was injected into the HPLC/Q-TOF MS system. Gallic acid (catalogue #91215, Sigma-Aldrich, St. Louis, MO, USA), paeoniflorin (catalogue #P0038, Sigma-Aldrich), and paeonol (catalogue #H35803, Sigma-Aldrich) were separately prepared in 100 *μ*L of blank sera to obtain concentrations of 20.0, 10.0, and 40.0 ng/mL, respectively. The spiked sera were prepared as mentioned above for the LD sera and were analysed by the same system.

The HPLC was a ThermoFisher ACCELA LC system. The mobile phase consists of a mixed aqueous solution of methanol-ammonium acetate (5 mM). The flow rate was set at 0.3 mL/min. The column temperature was maintained at 30°C. Detection was achieved using a ThermoFisher TSQ Quantum Ultra Triple-Quadrupole Mass Spectrometer with an electrospray ionization (ESI) interface in the negative-ion mode. The parameter settings were as follows: spray voltage, 2,000 V; capillary temperature, 400°C; sheath gas (nitrogen), 40 arbitrary units; auxiliary gas (nitrogen), 10 arbitrary units; vaporizer temperature, room temperature. Detection of the compounds was performed in the selected-ion monitoring (SIM) mode with *m*/*z* 169.0 [M − H]^−^ for gallic acid, *m*/*z* 479.4 [M − H]^−^ for paeoniflorin, and *m*/*z* 167.0 [M − H]^−^ for paeonol. ThermoFisher LCQUAN software was used to analyse the chromatograms.

### 2.5. Cytotoxicity Assay

The ability of the rat medicated sera containing GFW to potentiate cisplatin or paclitaxel (Hisun Pharmaceutical Co., Ltd., Zhejiang, China) cytotoxicity was evaluated in SKOV3/DDP and SKOV3 cells using cell counting kit-8 (CCK-8) (Sigma-Aldrich, St. Louis, MO, USA) assay [26]. Briefly, the cells were harvested in the exponential growth phase and were seeded into 96-well plates at a density of 1.0 × 10^4^ cells per well. The supernatant was removed after the cells adhered to the wall, and then FBS-free RPMI 1640 medium was added followed by incubation for another 24 hours at 37°C. Additionally, the cells were treated with the rat medicated control sera, the LD, MD, and HD sera, the LD sera, or verapamil (VER, Harvest Pharmaceutical Co., Ltd., Shanghai, China; 5 *μ*mol/L) plus cisplatin or paclitaxel. The sera volume ratio for incubation is 10%. After incubation at 37°C for 72 hours, WST-8 [2-(2-methoxy-4-nitrophenyl)-3-(4-nitrophenyl)-5-(2, 4-disulfophenyl)-2*H* tetrazolium, monosodium salt], which can be bioreduced by cellular dehydrogenases to an orange formazan product, which is then dissolved in cell culture medium, was added to the cells at a volume ratio of 1 : 10 to incubate for 2.5 hours. The optical density (OD) was measured at 450 nm using an enzyme immunoassay analyser (Dynatech MR4100, USA). Cell viability (% of control) was calculated as follows: (OD_test_ − OD_blank_)/(OD_control_ − OD_blank_) and growth inhibition rate was calculated as follows: 1 − cell viability%. The concentrations required to inhibit growth by 50% (IC_50_ values) were calculated from the cytotoxicity curves using Bliss's method. The resistance fold values, used as the parameter for resistance, were calculated by dividing the IC_50_ of SKOV3/DDP cells by the IC_50_ of SKOV3 cells to cisplatin or paclitaxel or other combinations as above. The reversal fold values, used as the potency parameter for reversal, were calculated by dividing the IC_50_ of cisplatin or paclitaxel alone by the IC_50_ of cisplatin or paclitaxel in combination with the LD sera or VER.

### 2.6. Flow Cytometric Assay of Rhodamine 123 Cellular Accumulation

The assay to analyse the function of P-gp in SKOV3/DDP cells was carried out using a modified method as previously described [27]. Briefly, the cells were adjusted to a density of 2 × 10^6^/mL, resuspended in serum-free medium, and distributed as 0.5 mL aliquots into 1 mL Eppendorf tubes. The LD, MD, and HD sera, VER (5 *μ*mol/L), and vehicle were added to the aliquoted samples, and the samples were incubated at 37°C for 24 h before rhodamine 123 (Rho123; Sigma-Aldrich, St. Louis, MO, USA) was added at a final concentration of 0.5 *μ*g/mL. The tubes were incubated further at 37°C for 1 h, and then the samples were transferred to 4 mL flow cytometry tubes and washed twice with ice-cold phosphate-buffered saline (PBS) before being resuspended in 0.5 mL of PBS. Flow cytometry analysis was carried out to evaluate the mean fluorescence intensity (MFI) of Rho123 in SKOV3/DDP cell using the EPICS XL-MCL flow cytometer (Beckman Coulter, Fullerton, CA, USA). All analyses were performed in duplicate in at least three separate experiments. The data were analysed using Expo32 ADC software (Beckman Coulter, Fullerton, CA, USA).

### 2.7. Western Blotting Assay

Western blotting analyses of P-gp, PI3K, AKT, phospho-AKT (p-AKT), and mTOR proteins were performed using a slight modification of the method described previously [28]. Briefly, SKOV3/DDP cells were seeded into 6-well plates at a density of 2.0 × 10^6^ cells per well and were treated with the rat medicated control sera, the LD, MD, and HD sera for 72 h, and then the cells were lysed using RIPA lysis buffer (Beyotime Institute of Biotechnology, China). The protein concentrations were determined using the bicinchoninic acid assay (BCA; Santa Cruz Biotechnology, USA). A total of 40 *μ*g of proteins was electrophoresed via sodium dodecyl sulphate polyacrylamide gel electrophoresis (SDS-PAGE) and transferred onto nitrocellulose membranes (Millipore, USA), which were then blocked with 5% skimmed milk powder for 1 hour at room temperature. The membranes were next incubated with rabbit polyclonal antibody to PI3K p110*α* (clone H-201, catalogue #sc-7174, 1 : 500 dilution; Santa Cruz Biotechnology), AKT (clone H-136, catalogue #sc-8312, 1 : 1000 dilution; Santa Cruz Biotechnology), p-AKT (Ser473, catalogue #sc-33437, 1 : 1000 dilution; Santa Cruz Biotechnology), mTOR (clone H-266, catalogue #sc-8319, 1 : 1000 dilution; Santa Cruz Biotechnology), or mouse monoclonal antibody to human P-gp (catalogue #sc-55510, 1 : 500 dilution; Santa Cruz Biotechnology) and *β*-actin (catalogue #sc-47778, 1 : 500 dilution; Santa Cruz Biotechnology) at 4°C overnight. Following primary antibody incubation, the membranes were washed with TBST, and goat anti-rabbit IRDye 800CW (926-32211, 1 : 5000 dilution) or goat anti-mouse IRDye 680LT secondary antibody (926-68020, 1 : 10000 dilution; Li-COR Biosciences, NE, USA) was added, respectively. After incubation at room temperature for 2 hours, the bands were detected using the Odyssey Infrared Fluorescent Western Blots Imaging System from Li-COR Bioscience (Lincoln, NE, USA). The optical density of each band was measured with a computer-assisted imaging analysis system (Quantity One, Bio-Rad, Hemel Hempstead, UK) and the relative protein levels were normalized to optical density of *β*-actin.

### 2.8. *In Vivo* Tumour Xenografts and Reversing Efficacy Test

The ability of GFW to potentiate the antitumour activity of cisplatin/paclitaxel was evaluated using the SKOV3/DDP xenografts as described previously [29]. Briefly, female BALB/c nude mice, aged between 3 and 4 weeks and weighing 20~22 g, were housed in standard microisolator conditions free of pathogens and used in accordance with the Animal Care and Use protocol approved by Nanyang Institute of Technology. Next, 2 × 10^7^ SKOV3/DDP cells were injected into the left flanks of each mouse selected for the experiment. When the tumours reached a diameter of circa 6 mm (approximately 116 mm^3^), the mice were randomly divided into different treatment groups comprising 10 mice in each group: a control group (normal saline administered orally and consecutively for 21 days), a single LD GFW group at dosage of 4 g·kg^−1^·d^−1^ administered orally for 21 days, a cisplatin (10 mg/kg administered intraperitoneally (i.p.) per 4 days) plus paclitaxel (15 mg/kg administered intravenously (i.v.) per 3 days) group, a combination of cisplatin, paclitaxel, and LD GFW group, and a combination of cisplatin, paclitaxel, and VER (5.0 mg·kg^−1^ administrated i.v. for 21 days) group. The animals were weighed, and the tumour length (*L*) and width (*W*) were measured every 4 days. The tumour volume (*TV*) was calculated according to published article [30] as follows: *TV* = 1/2 × *L* × *W* ^2^. After the last treatment, tumours were resected and weighted. The percent of tumour growth inhibition was calculated by comparing the average tumour weights of the treated groups with those of the tumour-bearing control group. Tumour growth in saline treated control animals was taken to be at 100%.

### 2.9. Statistical Analysis

For all of the experiments, the data are presented as the mean ± SD. Tests for significant differences between the groups were performed using one-way ANOVA with multiple comparisons (Fisher's pairwise comparisons) using SPSS 20.0. A minimum *P* value of 0.05 was estimated as the significance level for all tests. Graphic representations were performed using GraphPad Prism version 6.01 for Windows.

## 3. Results

### 3.1. Bioactive Constituents in Rat Medicated Sera after the Administration of GFW

**Figure 1:**
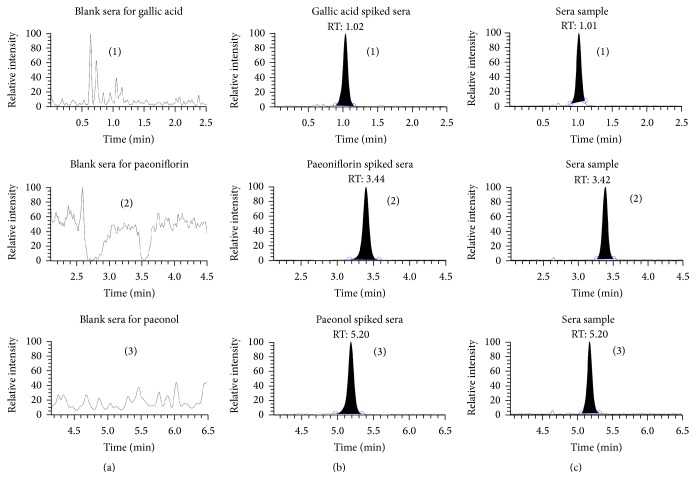
Typical chromatograms of HPLC/QqQ MS of gallic acid (1), paeoniflorin, (2) and paeonol (3). (a) Blank rat sera; (b) blank rat sera spiked with gallic acid, paeoniflorin, and paeonol; (c) rat sera sample obtained 30 min after the last oral administration of low-dose GFW.

We investigated whether the 3 potent MDR inhibitors, gallic acid, paeonol, and paeoniflorin in GFW, would be present in rat medicated sera after the administration of GFW. The chromatograms of the blank sera, spiked sera samples, and rat medicated sera samples after the oral administration of GFW are shown in Figure 1. Blank sera samples were screened and found to be free of interference from endogenous components or other sources at the same mass transitions (Figure 1(a)); the retention times (RTs) for gallic acid, paeonol, and paeoniflorin standards (Figure 1(b)) in the spiked sera samples were the same as those detected for three bioactive constituents in the rat medicated sera (Figure 1(c)), indicating that the 3 potent MDR inhibitors were present in the rat medicated sera.

### 3.2. *In Vitro* Reversing Effects

**Figure 2:**
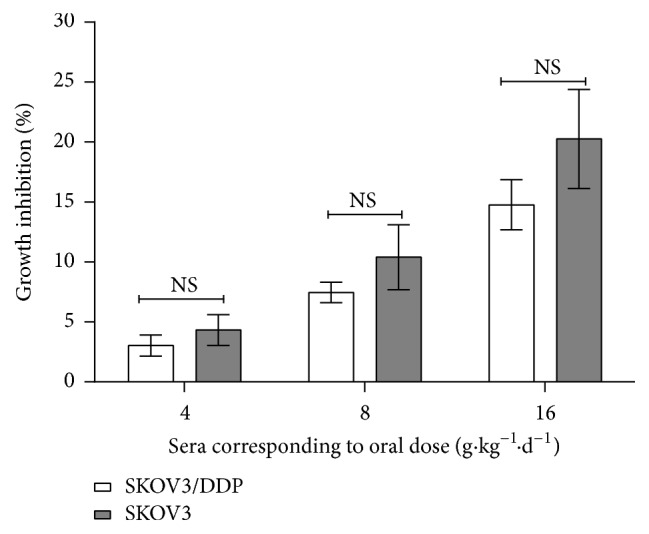
Effect of the rat medicated sera containing GFW corresponding to different oral dosages on the proliferation of SKOV3 and SKOV3/DDP cells. After treatment, the cells were washed and cultured for 72 h at 37°C in 96-well plates. The values correspond to the mean ± SD of three independent experiments. Significant differences between the means are specified by capped lines; NS indicates no significant.

The ability of the rat medicated sera containing GFW to reverse the resistance against SKOV3/DDP cells was investigated, and the LD sera were used to study the reversing effect because they have the lowest growth inhibition effect on SKOV3 and SKOV3/DDP cells (less than 5%, Figure 2). The resistance fold of SKOV3/DDP cells to cisplatin and paclitaxel was approximately 4.13 and 3.85 compared with that of the SKOV3 cells. In the drug-resistant SKOV3/DDP cells, coincubation of the LD sera or VER with cisplatin or paclitaxel enhanced significantly the cytotoxic effect of cisplatin or paclitaxel (*P* < 0.01, Table 1). However, there is no significant difference between the LD sera in combination with cisplatin or paclitaxel and VER in combination with cisplatin or paclitaxel, although the reversal fold of the former was lower than those of the latter. These results indicate that the LD sera have similar reversing effect with VER* in vitro*.

### 3.3. Flow Cytometric Assay of Rho123 Accumulation in SKOV3/DDP Cells

**Figure 3:**
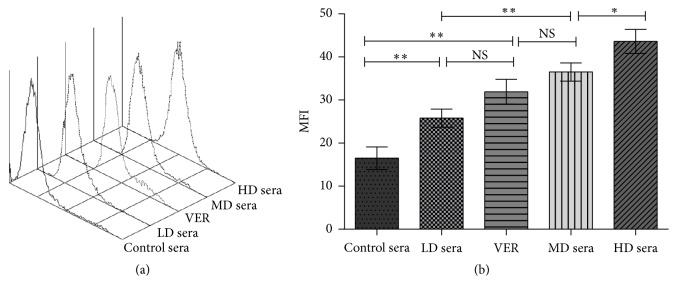
P-gp function changes in SKOV3/DDP cells after treatment for 24 h with verapamil or the rat medicated sera containing GFW corresponding to oral doses by flow cytometric analyses. (a) Representative flow cytometry overlay plot is shown. The P-gp inhibition effect increased along with the peaks moving far away from the vertical-axis. (b) Bar graph of mean fluorescence intensities (MFIs) of Rho123 in SKOV3/DDP cells with different treated groups. The results are the mean ± SD of 3 independent experiments. ^*∗∗*^ *P* < 0.01, ^*∗*^ *P* < 0.05; NS, no difference. LD sera represent the rat medicated sera corresponding to the GFW dosage of 4 g·kg^−1^·d^−1^. MD sera represent the rat medicated sera corresponding to the GFW dosage of 8 g·kg^−1^·d^−1^. HD sera represent the rat medicated sera corresponding to the GFW dosage of 16 g·kg^−1^·d^−1^.

The rat medicated sera containing GFW corresponding to different oral dosages were examined in a short-term Rho123 accumulation assay for their P-gp inhibitory effect on SKOV3/DDP cells. The results are displayed in Figure 3. The presence of the rat medicated sera containing GFW resulted in an increase in Rho123 accumulation in a dose-dependent manner in SKOV3/DDP cells, manifesting as peaks moving far away from the vertical-axis, and the MFI increased with the dose of the rat medicated sera. The effect of increasing Rho123 accumulation by the LD sera was similar to that of VER, manifesting the MFIs between them have no significant difference (*P* > 0.05). These results suggest that P-gp function was inhibited by the rat medicated sera containing GFW. In these short-term experiments, no signs of toxicity or cell damage were observed directly in microscope after stained with trypan blue.

### 3.4. GFW Disrupts the PI3K/AKT/mTOR Signalling Pathway

**Figure 4:**
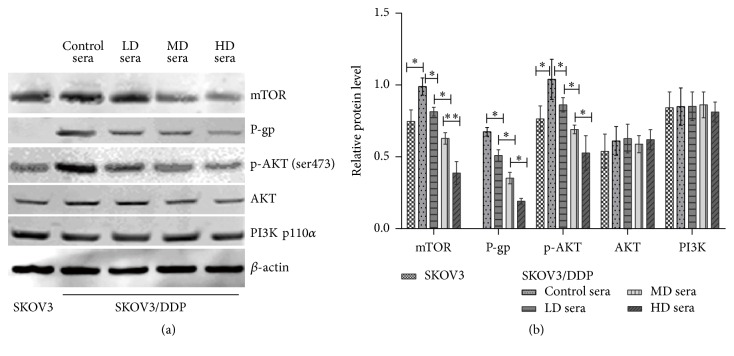
Rat medicated sera containing GFW inhibit MDR involved in P-gp and the PI3K/AKT/mTOR signalling pathway by Western blotting analysis in SKOV3/DDP cells after treated for 72 h. (a) Representative Western blotting gel results. (b) Bar graphs for relative protein levels. The results are the mean ± SD of at least 3 independent experiments. *β*-actin was used as an endogenous control and densitometric values were normalized by *β*-actin. ^*∗∗*^ *P* < 0.01, ^*∗*^ *P* < 0.05. LD sera represent the rat medicated sera corresponding to the GFW dosage of 4 g·kg^−1^·d^−1^. MD sera represent the rat medicated sera corresponding to the GFW dosage of 8 g·kg^−1^·d^−1^. HD sera represent the rat medicated sera corresponding to the GFW dosage of 16 g·kg^−1^·d^−1^.

We employed Western blotting to test whether the PI3K/AKT/mTOR signalling pathway was involved in the reversing effect of the rat medicated sera containing GFW. As shown in Figure 4, the levels of P-gp and p-AKT at Ser473 as well as the expression of mTOR, a known downstream target of AKT, in SKOV3/DDP cells, were increased significantly compared with those in SKOV3 cells (*P* < 0.05). After treated with the rat medicated sera containing GFW in SKOV3/DDP cells, the levels of P-gp, p-AKT, and mTOR were decreased in a dose-dependent manner (*P* < 0.01, *P* < 0.05). Whereas the levels of PI3K p110*α* and total AKT in SKOV3/DDP cells were not different compared with those in SKOV3 cells (*P* > 0.05), no changes were observed after SKOV3/DDP cells were treated with the rat medicated sera (*P* > 0.05). These results indicated that the sera inhibited MDR in SKOV3/DDP cells possibly through the downregulation of P-gp expression and the involvement of the PI3K/AKT/mTOR signalling pathway.

### 3.5. *In Vivo* Efficacy Evaluation

The ability of GFW to reverse MDR* in vivo* was tested in BALB/c xenograft nude mice generated with SKOV3/DDP cells. Compared with mice treated with saline, mice treated with a single LD of GFW showed no reduction in tumour growth and no death was observed with 21 days of administration. A similar outcome was obtained by measuring the weight of the mice and excised tumours at the end of the administration (*P* > 0.05). However, 2/10 mice died in the saline treatment group at the end of the administration; this may result from progression of disease. These results suggest that LD GFW has no apparent antitumour effect and may own the property of low toxicity on tested animals.

**Figure 5:**
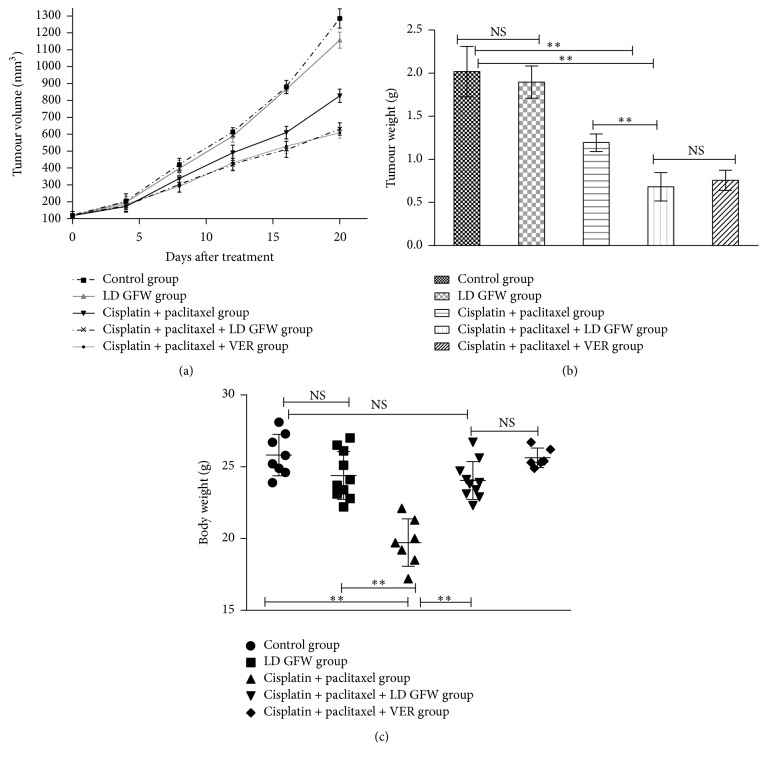
Inhibition of xenograft ovarian cancer by GFW. BALB/c nude mice bearing tumours formed by SKOV3/DDP cells were given saline as controls, a single GFW dosage of 4 g·kg^−1^·d^−1^(LD GFW), cisplatin (10 mg/kg administered intraperitoneally (i.p.) per 4 days) and paclitaxel (15 mg/kg administered i.v. per 3 days), combination of cisplatin, paclitaxel, and LD GFW, or combination of cisplatin, paclitaxel, and VER (5.0 mg·kg^−1^ administrated i.v. for 21 days) (*n* = 10). The figures show the average tumour volumes along with the days after different treatments (a), average excised tumour weights (b), and body weights (c) of mice at the end of observation. ^*∗∗*^ *P* < 0.01; NS, no difference.

The combination of cisplatin and paclitaxel showed tumour growth inhibition of 40.66% at the end of the administration in the resistant SKOV3/DDP xenograft tumours, and the average tumour volume was decreased significantly along with 21 days of administration, while 3/10 mice died, and the average weight of the mice was reduced significantly at the end of the administration compared with that in the mice treated with saline (*P* < 0.01); this may result from chemotherapeutic toxicities of cisplatin and paclitaxel. By contrast, no mice died, and the average weight of the mice showed no significant decrease at the end of the administration in the combination cisplatin, paclitaxel, and LD GFW group (*P* > 0.05); however, the tumour growth inhibition increased to 66.2% at the end of the administration compared with that in the mice treated with saline. In the meanwhile, a similar tumour growth inhibition effect was observed in the combinative treatment of cisplatin, paclitaxel, and VER group; however, also 4/10 mice died at the end of administration in this group (Figures 5(a), 5(b), and 5(c)). These* in vivo* findings demonstrated that LD GFW enhances the anticancer efficacy of cisplatin and paclitaxel treatment in resistant SKOV3/DDP xenograft tumours with mice body weights being maintained.

## 4. Discussion

In recent years, interest in the potential of plants and herbal medicine as a source of novel medicinal agents has grown significantly. Substantive research, aimed at utilizing this vast natural resource, is being carried out worldwide. In the development of anticancer agents, a wide range of plant-derived cytotoxic agents and/or MDR inhibitors were discovered from plant extracts. However, a large amount of time and effort is required before these agents can reach the market for clinical use due to insufficient evidence supporting their safety. For example, the development of camptothecins and taxanes as drugs for clinical use took over twenty years [31].

TCM has more than 2,000-year history of dealing with diseases and has evolved over time to become the second largest health care system in the world, after modern Western medicine. In general, treatment with Western medicine is not satisfactory for chronic diseases such as cardiovascular disease, type 2 diabetes mellitus, chronic pain, and cancer; TCM may offer an effective option [32, 33]. In addition, the long history of TCM use may dramatically reduce the time to its clinical use, if a TCM formula is found that has new therapeutic effects apart from its traditional usage. GFW is a TCM formula used in China, Japan, and other Asian countries that has been proven to be very safe and effective with fewer harmful side effects according to its long history of use. Recently, GFW was reported to improve the curative effect of chemotherapy with lower side effects when combined with cisplatin in OC patients during a 3-month treatment cycle, but the exact mechanism is unknown [34]. We suspect that enhancement of the effect may be involved in certain mechanisms delaying and/or overcoming MDR in chemotherapy. To address the possible mechanisms, a novel seropharmacology method arising recently for pharmacological study was adopted to study TCM* in vitro* using animal-medicated sera [35]. TCM formulas typically comprise more than 3 herbs, making their extracts contain hundreds or thousands of components; for example, GFW was found to contain 27 main components, including monoterpene glycosides, acetophenones, galloyl glucose, and even some isomers in its extract [36]. The essence of seropharmacology is to administer TCM to experimental animals (generally rabbits or rats), followed by harvesting the animal sera after a period of time. The sera are then applied to different cell lines for molecular mechanism studies. The advantage of seropharmacology for TCM study is the use of a TCM formula as a whole and the focus on the actual pharmaceutical ingredients or internal biological metabolic compounds of the formula, avoiding the isolation of individual components which may remove other potential key components that may not be detected using current technologies. We found that the 3 potent MDR inhibitors, gallic acid, paeonol, and paeoniflorin, in GFW extract were present in the rat medicated sera after the administration of GFW (Figure 1) by HPLC/QqQ MS technology.

The* in vitro* reversal effects of the rat medicated sera containing GFW were studied with first-line chemotherapeutical drugs for OC, cisplatin and paclitaxel, in SKOV3/DDP cells and its parental cisplatin-sensitive counterpart. We found that the sera containing GFW significantly restored the sensitivity of SKOV3/DDP cells to cisplatin and paclitaxel. However, in SKOV3 cells, the rat medicated sera containing GFW had no significant effect on drug cytotoxicity. Moreover, we showed that GFW could overcome the resistance of cisplatin and paclitaxel in xenograft tumour models generated with SKOV3/DDP cells (Figures 5(a) and 5(b)). The reversal effect of the rat medicated sera containing GFW was similar to those of the known MDR inhibitor, verapamil, which was popularly used as a positive control for research on inhibition of P-gp function [37, 38]. Since a median lethal dose of VER for the i.v. administration in mouse was reported to be 7.6 mg·kg^−1^ [39], a single i.v. dose of 2.5 mg·kg^−1^ VER still caused the death of 40% of animals in the* in vivo* efficacy test, which may result from the cardiotoxicity of VER [40]; instead, no mice died in the combinative treatment of cisplatin, paclitaxel, and LD of GFW group. These data suggest, for the first time, that GFW may be an efficacious reversal TCM formula for ovarian cancer* in vitro* and* in vivo*.

P-gp modulation is thought to be an effective strategy to reverse MDR by inhibiting P-gp function and/or expression [41], reducing the clearance of anticancer drugs by cancer cells and then dramatically increasing their toxicity. Rho123 has been used as a marker for detecting the changes in P-gp function due to its low toxicity and specificity for P-gp [42, 43]. Some authors have reported that Rho123 is a substrate for both MRP and P-gp [44], but it was also reported that Rho123 is transported approximately 10 times higher by P-gp than by MRP1, and it is useless for the test of MRP1 activity [45]. In this study, we demonstrated that Rho123 accumulation was increased in a dose-dependent manner after adding the rat medicated sera containing GFW in SKOV3/DDP cells, suggesting that GFW reverses MDR through the inhibiting of P-gp function.

The PI3K/AKT/mTOR pathway is crucial for the regulation of chemoresistance in various cancers [46]. Activated receptor tyrosine kinase (RTK) associates with the p85 SH2-domain-containing subunit and recruits and activates the p110 catalytic domain of PI3K. Activated PI3K phosphorylates membrane phosphatidylinositol-4,5-bisphosphate (PIP2) in the 30 position, which acts as a docking site for phosphoinositide-dependent kinase 1 (PDK1) and AKT. AKT is activated by PDK and regulates downstream effectors, such as* MDR1*, which regulates drug resistance [21, 47]. mTOR participates in cell survival and proliferation, in part, through its ability to control AKT activity by the phosphorylation of AKT at Ser473 [48]. The inhibition of AKT phosphorylation could significantly downregulate the expression of P-gp and thus partially reverse MDR [49]. Due to its mechanistic activity involving these crucial pathways that directly or indirectly interplay with P-gp activities, we posed a potent functional effect for GFW to affect such interaction. Our results demonstrated that the rat medicated sera containing GFW could inhibit the protein levels of P-gp, p-AKT at Ser473 and mTOR, which were significantly different between SKOV3/DDP cells and their parental cisplatin-sensitive counterpart, in a dose-dependent manner in SKOV3/DDP cells, while no effect has been shown on the protein levels of PI3K p110*α* and total AKT, which were not significantly different between SKOV3/DDP and their parental cisplatin-sensitive counterpart, suggesting that the effect of MDR reversal by GFW may occur through inhibition of the activated PI3K/AKT/mTOR signalling pathway.

In summary, this study demonstrates, for the first time, the reversal MDR potential of GFW for ovarian cancer* in vitro* and* in vivo* by inhibiting the protein expression and function of P-gp, which may act through the inactivation of the PI3K/AKT/mTOR signalling pathway. The combination of cisplatin and/or paclitaxel and GFW may provide a therapeutic benefit against cisplatin-resistant ovarian cancers after further validation.

## Figures and Tables

**Table 1:** Cytotoxicity of the LD sera or VER in combination with cisplatin or paclitaxel in SKOV3 and SKOV3/DDP cells* in vitro*.

Drug and concentration	IC_50_ (mg·L^−1^)		Resistance fold	Reversal fold
	SKOV3	SKOV3/DDP		
Cisplatin	3.58 ± 0.12	14.79 ± 0.41^*∗∗*^	4.13	
Paclitaxel	1.78 ± 0.31	6.86 ± 0.22^*∗∗*^	3.85	
LD sera + cisplatin	3.22 ± 0.08	4.39 ± 0.17^*∗∗*^	1.36	3.04
LD sera + paclitaxel	1.59 ± 0.27	2.33 ± 0.42^*∗∗*^	1.47	2.62
VER + cisplatin	3.34 ± 0.13	4.09 ± 0.29^#^	1.22	3.39
VER + paclitaxel	1.69 ± 0.24	1.95 ± 0.30^#^	1.18	3.26

LD, low-dose. VER, verapamil. ^*∗∗*^ *P *< 0.01 compared with each other, ^#^ *P *< 0.01 compared with each other.
